# Physical Activity as a Mediator in the Relationship Between Race or Ethnicity and Changes in Multimorbidity

**DOI:** 10.1093/geroni/igaa057.1098

**Published:** 2020-12-16

**Authors:** Jason Newsom, Emily Denning, Ana Quinones, Miriam Elman, Anda Botoseneanu, Heather Allore, Corey Nagel, David Dorr

**Affiliations:** 1 Portland State University, Portland, Oregon, United States; 2 Oregon Health & Science University, Portland, Oregon, United States; 3 OHSU-PSU School of Public Health, Portland, Oregon, United States; 4 University of Michigan, Dearborn, Michigan, United States; 5 Yale University, New Haven, Connecticut, United States; 6 University of Arkansas for Medical Sciences, Little Rock, Arkansas, United States

## Abstract

Racial/ethnic disparities in multimorbidity (≥2 chronic conditions) and their rate of accumulation over time have been established. Studies report differences in physical activity across racial/ethnic groups. We investigated whether racial/ethnic differences in accumulation of multimorbidity over a 10-year period (2004-2014) were mediated by physical activity using data from the Health and Retirement Study (N = 10,724, mean age = 63.5 years). Structural equation modeling was used to estimate a latent growth curve model of changes in the number of self-reported chronic conditions (of nine) and investigate whether the relationship of race/ethnicity (non-Hispanic Black, Hispanic, non-Hispanic White) to change in the number of chronic conditions was mediated by physical activity after controlling for age, sex, education, marital status, personal wealth, and insurance coverage. Results indicated that Blacks engaged in significantly lower levels of physical activity than Whites (b = -.171, □ = -.153, p < .001), but there were no differences between Hispanics and Whites (b = -.010, □ = -.008, ns). Physical activity also significantly predicted both lower initial levels of multimorbidity (b = -1.437, □ = -.420, p < .001) and greater decline in multimorbidity (b = -.039, □ = -.075, p < .001). The indirect (mediational) effect for the Black vs. White comparison was significant (b = .007, □ = .011, 95% CI [.004,.010]). These results provide important new information for understanding how modifiable lifestyle factors may help explain disparities in multimorbidity in middle and later life, suggesting greater need to reduce sedentary behavior and increase activity.

